# Cell-Penetrating Peptides (CPPs) as Therapeutic and Diagnostic Agents for Cancer

**DOI:** 10.3390/cancers14225546

**Published:** 2022-11-11

**Authors:** Ryan A. Bottens, Tohru Yamada

**Affiliations:** 1 Department of Surgery, Division of Surgical Oncology, College of Medicine, University of Illinois, Chicago, IL 60612, USA; 2 Richard & Loan Hill Department of Biomedical Engineering, College of Medicine and Engineering, University of Illinois, Chicago, IL 60607, USA

**Keywords:** cell penetration, protein transduction, targeted delivery, therapeutics, diagnostics, clinical trial

## Abstract

**Simple Summary:**

Crossing biological barriers is often required for drug delivery. Cell-penetrating peptides (CPPs) are short strands of amino acids that have been widely used as a delivery vehicle to overcome barriers for various applications. This review aims to emphasize the role of CPPs as permeation enhancers for targeted drug delivery applications. We also discuss the prospect of clinical translation of CPP-functionalized drug delivery systems in oncology. This may help facilitate the development of new types of CPPs for preventing or treating cancer.

**Abstract:**

Cell-Penetrating Peptides (CPPs) are short peptides consisting of <30 amino acids. Their ability to translocate through the cell membrane while carrying large cargo biomolecules has been the topic of pre-clinical and clinical trials. The ability to deliver cargo complexes through membranes yields potential for therapeutics and diagnostics for diseases such as cancer. Upon cellular entry, some CPPs have the ability to target specific organelles. CPP-based intracellular targeting strategies hold tremendous potential as they can improve efficacy and reduce toxicities and side effects. Further, recent clinical trials show a significant potential for future CPP-based cancer treatment. In this review, we summarize recent advances in CPPs based on systematic searches in PubMed, Embase, Web of Science, and Scopus databases until 30 September 2022. We highlight targeted delivery and explore the potential uses for CPPs as diagnostics, drug delivery, and intrinsic anti-cancer agents.

## 1. Introduction

The selective semi-permeability of the cell membrane protects the cell interior from harsh materials from outside of the cell while also allowing vital nutrients and materials to pass in and out. This same mechanism presents a real challenge when trying to deliver selective molecules into the cell. Current attempts to deliver therapeutics and diagnostic agents into cells are arduous and prone to restrictions and errors [1,2]. Despite extensive research efforts on drug delivery, it generally yields low cell specificity and leads to significant toxicity [1,2]. The ideal goal is to deliver the cargo molecules to the desired cancerous cells and avoid the healthy cells. Cell-penetrating peptides (CPPs) or protein transduction domains (PTDs) are relatively short and, in many cases, cationic amino acids that possess the titular ability to penetrate the cellular membrane [2]. Some CPPs can selectively interact with target cells with high accuracy and efficiency, and operate even at low concentrations. Several modes of CPPs internalization have been discussed [3,4,5,6,7]. Moreover, one of the most attractive aspects of CPPs is their ability to covalently link to macromolecular cargos, such as DNA, RNA, and proteins and deliver them into the cell. Thus, larger biomolecules that would normally be restricted from entering the cell, because of the selective impermeability of the cell membrane, can be translocated into the cell while being escorted by CPPs. Early indicators suggest that some of CPPs have a higher uptake efficacy and delivery efficiency while remaining less cytotoxic than other similar treatments, such as nanoparticles or virus vectors [8].

Since their discovery in 1988, the high potential and variety of CPPs have led to scores of different peptides being cataloged, all with different specificity and cargo-carrying capabilities. In recent decades, upwards of 1850 CPPs have been cataloged within CPPsite 2.0 [9], each with a unique peptide sequence, target cell specificity, and covalent cargo binding capabilities [10]. For example, the Trans-activator of Transcription (TAT) protein identified from the HIV1 virus allows for penetration of the cellular membranes and allows for direct interaction with the cell nucleus [11]. The chimeric approaches have been made to combine known CPPs such as TAT, Transportan, or pVEC with novel peptides [12,13,14,15,16,17,18,19]. All this is to highlight that the discovery and further research into the domain of CPPs yield countless possibilities on how to attack cancer by targeting specific intracellular molecules or organelles, whether it be by disrupting oncogenic pathways as a preventative measure, or by introducing therapeutic or diagnostic agents to cancer cells. In this review, we highlighted and summarized the recent updates of CPPs-based targeted delivery along with the therapeutic and diagnostic potential.

## 2. Types of CPPs

CPPs achieve a wide range of uses and cargo capabilities, in part, because there is a vast array of peptides to choose from, offering case-by-case specifications for CPP-based therapeutics. There are several ways to classify and categorize CPPs and in this review, we describe the different types of CPPs based on their physical characteristics, specifically charge.

### 2.1. Cationic CPPs

At physiological pH, cationic CPPs yield a net positive charge and show great affinity at being able to penetrate the cell and circumvent the need to interact with the cell through receptors [10]. The property of certain molecules’ intrinsic ability to penetrate the cell membrane better than others was noticed as early as 1965 when cationic polymers such as polylysine and polyarginine, induced significantly higher cellular uptake of albumin by cultured cancer cells [2]. Since then, numerous comparisons have been made differentiating the uptake potential of positively charged, short peptide chains against their long or net-neutral counterparts. Specifically, it was shown that short homopolymers of arginine (R) had higher uptake compared to other amino acids including polylysine chains [20,21,22,23]. Attempts to utilize and mimic this ability have been undertaken, achieving excellent results. Different types of peptides present certain advantages or disadvantages over others and each utilizes differing pathways for cellular penetration. Arginine is one of the few cationic natural amino acids (pKa > 12) and possesses the ability to interact with negatively charged integrated proteins and induce translocation into the cell, carrying with it the specified cargo [24]. The uptake efficacy increases with arginine length, but beyond 8 to 10 arginine residues, while translocation can occur, but it may result in damage to the membrane, possibly inhibiting future CPP applications [24]. For instance, pVEC (LLIILRRRIRKQAHAHSK) [25] has multiple positively charged lysine and arginine amino groups, as well as the HIV-1-derived TAT protein (RKKRRQRRR) which also possesses these same characteristic arginine residues [26] (Table 1).

### 2.2. Amphipathic CPPs

Amphipathic CPPs contain a combination of polar and nonpolar amino acid residues. The nonpolar residues such as alanine (A), leucine (L), isoleucine (I), glycine (G), and valine (V) can interact with the nonpolar lipid head groups and insert the polar region into the membrane [1]. Among cataloged CPPs, those with amphipathic properties are the most common. Peptides such as ELP (VPGXG)_n_ where X is valine (V), alanine (A), or glycine (G) [70,71]; this peptide works as an Elastin-like protein (ELP) and is proposed to use as a hyperthermic approach against cancer [72,73]. Most naturally and synthetically occurring CPPs utilize the differences in polarity to infiltrate the cell through the membrane. 

CPPs mimicking the properties of naturally occurring CPPs to create more efficient and specific synthetic proteins have been designed. These CPPs are also combined with either natural or synthetic peptides, which can guide the CPP, along with its desired cargo, to the cell where it is needed. For instance, a chimeric combination between CPP pVEC (LLIILRRRIRKQAHAHSK-NH_2_) and a peptide designed to target glioma was successfully used to introduce fluorescent indicators into the cell [74]. The glioma homing peptide or gHo (NHQQQNPHQPPM-NH_2_) was combined with FAM (5-carboxyfluorescein) a fluorescent tag, which was identified to be able to translocate the tag cargo into the glioma [75]. Examples of CPPs that take advantage of this ability are Pep1 (KETWWETWWTEWSQPKKKRKV), which is a combination of a nonpolar amino group, and the NLS SV40 (Nuclear locating sequence) [39]. These types of combinations between the penetrating tail and the locating sequence are the basis of all CPP target specificity. 

### 2.3. Anionic CPP, p28, A Fragment of Azurin 

Another distinct category of the amphipathic CPPs is the negatively charged anionic CPPs. These peptide chains target and enter the cells differently than their cationic counterparts. A CPP, p28, has been found to have an ability of cancer preferential entry [59,60,61,62,63,64,76,77,78]. p28 is an anionic peptide made up of 28 amino acid residues (LSTAADMQGWTDGMASGLDKDYLKPDD) [65,66,79,80,81,82]. Specifically, the residues between Leu_50_-Asp_77_ within a protein known as azurin that are secreted by an opportunistic pathogen, *Pseudomonas aeruginosa* [67,68,83]. Azurin has been extensively studied as an electron transfer protein but has found itself to be the subject of many studies in the last few years due to its intrinsic ability to track down and enter cancer cells. The p28 region, a fragment of azurin, forms an alpha helix and interacts with beta microdomains called lipid rafts, which are generally overexpressed in cancer cells, along the cellular membrane and enter the cell.

## 3. Intracellular-Targeted Delivery by CPPs

**Figure 1:**
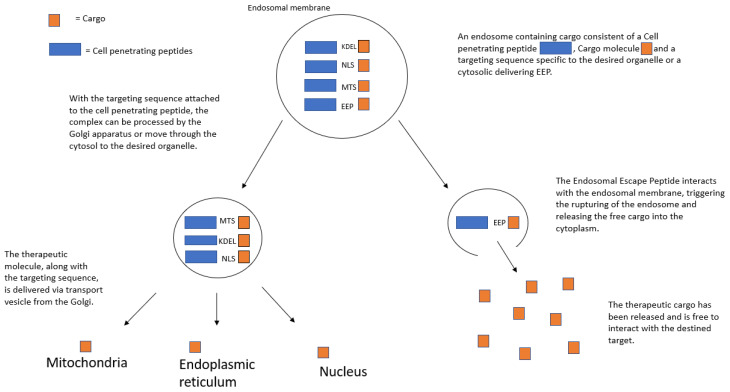
CPP-guided organelle targeting.

Particularly powerful characteristics of CPPs are the ability to not only penetrate the cellular membrane but also home in on certain organelles to increase the precision of target site specificity. Targeting specific organelles within the cell when it comes to fighting cancer is important due to the fact that targeted delivery to the specific intracellular targets can result in enhanced therapeutic efficacy and reduced toxicity [84,85,86,87,88]. Combining a specific organelle-locator sequence to the CPP, along with the therapeutic cargo, results in even greater control over the delivery of anti-cancer drugs. In this section, we summarize the different types of organelle-targeting strategies, as well as their current and potential future, uses in cancer therapeutics (Figure 1).

### 3.1. Nuclear Localization Signal (NLS)

The nucleus is the membrane-bound organelle that houses the cell’s complete genetic makeup in the form of DNA. Given that many genetic diseases originate from an error(s) in the genetic code, efforts are underway to target the nucleus more precisley. While the aforementioned HIV1 derivative, TAT, possesses an intrinsic ability to target the nucleus [89], better uptake rates can be achieved by combining the CPP with a nuclear-locating sequence. Interaction with the nucleus is achieved through the nuclear pore complex (NPC), which normally prohibits large molecules from entering the nucleus. Using an NLS, which can assist the cargo molecule, induces active transport through the nuclear pore, where the administration of the desired drug can then be achieved. For example, an NLS derived from the Simian virus antigen (SV40) can be covalently bound to both a CPP such as TAT and a DNA-repairing cargo to attack the disease at its genetic source [89]. Another study showed that a conjugation between a viral gene vector, adenovirus, and CPP-PEG resulted in an uptake increase into the nucleus by nearly 80 times when compared to the adenovirus alone [90] The combination of specified nuclear targeting sequences with their respective CPP shows great potential in the ability to not only penetrate the normally impermeable nuclear membrane, but also efficiently administer drugs or gene editing molecules to tackle the disease at the source.

### 3.2. Mitochondrial Targeting

The mitochondria are an integral part of the survival and overall health of the cell biome, as it is in charge of producing cellular energy in the form of ATP as well as being involved in cellular signaling pathways [91,92]. For these reasons, the mitochondria are an attractive target for therapeutics as disorders within the mitochondria can result in neurodegenerative or cancerous disease down the line. Mitochondrial-specific delivery is achieved by employing a mitochondrion-targeting sequence (MTS) with CPPs to deliver therapeutics or diagnostic dyes into the mitochondrial matrix. When the MTS is bound to the carboxy terminus of CPPs, the whole complex is processed by the mitochondrial membrane and is delivered into the matrix [93,94,95,96]. A common MTS is a positively charged lipid, triphenyl-phosphonium, or TPP, which uses its charge to interact with the anionic mitochondrial membrane, allowing for entry into the organelle [94]. Another example of a specific MTS combination (MLRAALSTARRGPRLSRLL) was bound to H_3_R_8_, a CPP, and was successful in delivering 5-FAM dye into the mitochondria. The dye itself is normally impenetrable to the mitochondrial membrane and is delivered better than when compared to the MTS dye complex alone [89]. 

### 3.3. Endoplasmic Reticulum Targeting

The folding of the endoplasmic reticulum (ER) is important to maximize the organelle’s surface area and volume to carry out the main ER functions which are protein folding and biosynthesizing lipids [89]. Since these functions are critical to biochemical pathways within the cell, disorders in the ER can lead to a myriad of diseases, including cancer. Proteins formed by ribosomes reach the ER either by the ribosome being embedded in the organelle or by being trafficked there by utilizing receptors on the outer ER membrane [97,98]. The receptors (KDEL-R) bind with a short locator sequence called KDEL (Lys-Asp-Glu-Leu) to allow synthesized proteins into the cell [99]. By using KDEL, ER-targeting CPPs have been created, and gold nanoparticles (AuNP) bound to the KDEL sequence showed a targeted localization at the ER [99]. 

### 3.4. Lysosomal Targeting

The lysosomal function consists of cellular waste degradation, and apoptosis, as well as playing a key role in intracellular signaling pathways [100,101]. Their involvement with apoptosis makes the lysosome an attractive target for cancer treatments. One way of granting lysosomal targeting is to use Lysosome Sorting Peptides (LSPs), usually short motifs consistent with tyrosine [102]. For example, a short LSP known as YXXO (Y being tyrosine, X being any amino acid residue, and O being any large hydrophobic group such as phenylalanine or isoleucine, among others.) has been shown to greatly increase uptake efficacy into the lysosome by interacting with the adapter protein complexes that make up the lysosome [103]. The use of these LSP-CPP conjugations warrants more research and trials but these studies suggested the possibility of controlled apoptotic treatments utilizing the lysosome intrinsic abilities [102].

### 3.5. Cytoplasmic Targeting through Endosomal Escape Peptides

The ability to influence over the cytoplasm can prove to be a useful tool. While organelle targeting can be achieved by manipulating the natural intracellular transport mechanisms, such as the Golgi complex or chaperone proteins, cytoplasmic targeting requires a different approach. Once CPP induces endocytosis to gain entry into the cell, the large impermeable cargo molecule is generally trapped within the endosome [104]. Endosomal escape efficiency has remained a barrier and the rate-limiting step for delivering intracellular cytosolic therapeutic proteins [104,105]. The ability of these large biomolecules to breach the endosomal membrane to reach their target is a critical step in intracellular therapeutics and has caused the search for and use of Endosomal Escape Peptides (EEPs) to assist the delivery.

EEPs are a class of conjugate peptides that can be used to disrupt the endosomal lining from within, thus triggering the release of the cargo molecule into the cytoplasm [104]. Molecules such as chloroquine have been bound to cargo molecules as a way of flooding the endosome with water, thus rupturing the endosomal lining [106]. Attempts to utilize an EEP-TAT-PTD consistent with indole and aromatic rings induce endosomal escape through their highly hydrophobic properties interacting with the endosomal membrane while having a less cytotoxic effect [105]. Likewise, domains such as E5TAT, HA2 (isolated from influenza), and ZF5.3 (a derivative of an avian pancreatic domain) have been bound to luciferase and tested to find their relative escape efficacy when compared to the luminescent cargo itself [104]. Conducting a reliable test to determine the relative concentrations of the delivered cargo is difficult, due to the dilution of the luminescent cargo within the cytoplasm. With this considered, it is proposed that the inclusion of EEPs can increase cytosolic delivery by a rate of 7 to 30 times greater than when compared to the cargo alone [104]. While this means that a large quantity of the specified cargo molecules will remain trapped within the endosome, this is an important step in finding a more efficient and reliable method of cytosolic targeting.

Attempts have been made to further increase the rate at which cargos are released from the endosome into the cytoplasm. While the use of EEPs greatly increases the rate at which the CPP complex is released compared to the CPP alone, it still falls short of optimal release rates. A possible solution to this issue is the use of multivalent CPPs (MCCPs), which essentially introduce a higher concentration of the CPP by adding multiple copies of the peptide to increase the interaction of the endosome [6,107]. The results showed that the uptake and delivery rates of MCCPs are comparable to that of regular CPPs but include the additional benefit of achieving endosomal escape more easily [6]. Another attempt at increasing endosomal escape rates is the use of pH-dependent membrane-active peptides (PMAPs). PMAPs work in a similar way to the HA2 virus in that they breach the endosomal membrane by increasing the pH within the lumen [108]. One peptide that can achieve that result is named GALA (WEAALAEALAEALAEHLAEALAEALEALAA) [109]. In general, these peptides contain large hydrophobic groups such as leucine or alanine along with groups such as aspartate or glutamate. The interaction between these amino acid residues releases protons into the lumen, acidifying the endosome and triggering endosomal escape [109].

## 4. Potential Diagnostics and Therapeutics

It has been suggested that there is great potential for the use of CPPs in clinical trials to treat cancer. The manipulability of CPPs provides new opportunities to precisely control the cell biome through transmembrane translocation, and the beginning looks promising for the future of CPP treatments. The ability to covalently link self-assembly CPPs and carry large, normally impermeable cargo molecules makes CPPs great tools with new abilities to deliver therapeutic medicines or imaging molecules into normally difficult-to-penetrate environments [2,110]. Traditionally, cancer cells presented a challenge simply getting into the cell, let alone manipulating it in any way because of some altered membrane expression. This especially applies to brain cancers such as glioblastomas, because while they are within the already impermeable cancer cell membrane, they are also protected by the blood-brain barrier (BBB), making CPPs such as novel p28 treatments so attractive because of their inherent permeability through the BBB and the cell membrane.

### 4.1. Imaging Tools and Diagnostics

As stated earlier, the covalent bonding of cargo molecules is the main attraction of CPPs and this has numerous possibilities when it comes to being able to manipulate the expression of cancer at a cellular level. The translational example of this potential is the ability to deliver bioluminescent molecules into cancer cells for diagnostic imaging and image-guided surgery. For instance, a difficulty, especially on the surgical front, is trying to precisely remove the tumor regions but not necessarily being able to fully differentiate between healthy and cancerous tissues without any image guidance [76,111]. This poses a challenge for surgeons aiming to both remove as much of the cancerous tissue as possible without affecting the surrounding normal tissues. For this purpose, ICG, a US FDA-approved nontoxic dye conjugated with a tumor-targeting CPP p28 (also known as ICG-p28) was created [59,60,76,77]. When viewed under near-infrared light wavelength, systemically administrated ICG-p28 provides a clear visualization of various types of tumors and identifies tumor margins that need to be removed surgically [59,60,76,77]. Removing the entire tumor region is critical for surgical cancer treatment, and the use of ICG-p28 aids in the visualization of hard to see deep tumor regions and can help prevent reproliferation [59,60,76,77]. The high tissue penetration, visibility, and differentiability of ICG-p28 mean that it is a prime candidate to be tested in clinical settings.

As cancer diagnostic tools, CPPs have also been proposed to deliver contrast agents for magnetic resonance imaging (MRI) [112,113,114,115], single photon emission computed tomography (SPECT) [116,117,118], positron emission tomography (PET) [119,120,121], and optical imaging [122,123]. CPPs have been chemically conjugated to contrast agents (e.g., metals, fluorescent, and radioactive materials) with the aim of more favorable pharmacokinetics/ biodistributions for cancer diagnosis [124]. In addition, to improve the tumor-specificity of CPPs, activatable CPPs have been proposed for more precise tumor visualization [10,125,126,127,128]. As CPPs are peptides that can be substrates for endogenous proteases. The activatable CPPs contain an amino acid sequence that can be targeted by matrix metalloproteases-2 (MMP-2) and MMP-9. Such metalloproteases are generally overexpressed in cancer cells [129,130]. When activated by MMP-2 and MMP-9, the targeted sequence within CPPs is cleaved, and their cellular uptake by tumor becomes greater [131]. There have been several attempts to design new activatable CPPs that can be activated by enzymatic activity, pH gradient, reactive oxygen species (ROS), and optical light. These approaches are summarized in a recent article [131]. 

Another promising area of development in CPPs to further increase their specificity is to use different combinations of transport domains such as nanoparticles. With further cell specificity, more detailed and reliable images of the tumor region can be possibly achieved. Dual targeting or dual-modality imaging binds nanoparticles with CPPs and they deliver the imaging agent jointly into the target cell [132]. Breast cancer cells treated with the dual imaging combination of CPP and NP yielded the highest concentration of DiR fluorescent dye when compared to CPP, NP, or DiR alone [133].

### 4.2. Anticancer Therapeutic Uses

Some CPPs have intrinsic anti-tumor properties along with cell-penetrating abilities. For example, p28, a fragment of *Pseudomonas* azurin protein, carries anticancer activity, along with the ability to enter tumor cells [61,63,64,65,66,76,77]. In preclinical testing, the anti-tumor efficacy of p28 was assessed on various types of human cancer cells such as brain, breast, prostate cancer, and melanoma. Upon cellular entry, p28 binds to a hydrophobic region within the DNA-binding domain of p53 and inhibits proteasomal degradation via an HDM2-independent pathway [64,65,66,134,135,136,137,138]. This results in an increase in the intracellular levels of p53 as well as its DNA-binding activity and elevates the cyclin-dependent kinase inhibitors, p21 and p27, thereby inducing cancer cell cycle arrest at G_2_/M and tumor growth inhibition [64,66].

The intrinsic anti-tumor properties of CPPs when paired with selected anticancer therapeutic cargos, also allow us to attack cancer at multiple levels. The specific therapeutic agent can be chosen based on circumstances in that specific patient and as the catalog of CPPs grows. For example, a synthetic CPP RLWMRWYSPRTRAYGC has been shown to disrupt tumor progression in lung cancers [5,139]. The innate anti-cancer effects of specific CPPs can be combined with the appropriate locating sequence and a chosen therapeutic to create a truly powerful tool against cancer. 

Given that some CPPs lack tumor-specificity which could lead to drug delivery to cells in healthy tissues, there have been several attempts to enhance the specificity of CPPs-based delivery by taking advantage of the natural targeting abilities of antibodies and antigens [140,141,142,143,144]. For instance, by linking the antibody against tumorous cells’ highly expressed antigens to a CPP-siRNA complex, siRNA gene-drug delivery can be achieved with high levels of specificity and little cytotoxicity [145]. While some CPPs have intrinsic tumor-targeting capabilities, the CPP–antibody conjugation allows for more general CPP use by utilizing the highly overexpressed antigens present on the surface of cancer cells [145,146]. This principle was tested in a preclinical prostate cancer animal model, in which there is almost universal overexpression of the prostate-specific membrane antigen (PMSA) [147]. By linking the PMSA antibody to a CPP bound with an siRNA, specifically TRIM24, a significant reduction in proliferation and colony formation was achieved [147]. However, antibody-based targeting does come with a challenge, which is controlling the release of the siRNA therapeutic once the complex has reached the desired destination [145]. Despite this drawback, there is still promise for antibody-CPP based drug delivery. 

In this section, we summarized the different types of CPP-based therapeutic and diagnostic strategies. CPPs have immense potential in both cancer diagnostic and therapeutic applications. CPPs are promising tools to improve cellular uptake which is one of the major contributions to developing an effective cancer treatment. Further basic/translational studies and clinical trials will provide a better understanding of the mechanisms involved in developing CPP-based cancer treatment.

## 5. Clinical Trials Utilizing CPPs

Previous preclinical studies suggest promising results for CPP-based treatments and diagnostics, not just in the domain of cancer, but across various types of disease treatment. Trials utilizing CPPs have been conducted to test the efficacy of CPP-based treatments for cancer therapeutics and diagnostics, and have yielded positive results, further showing the wide range of possibilities for future treatments. Here, we highlight CPPs in the clinical studies (Table 2). 

### 5.1. RI-TAT-p53C’ Trial

RI-TAT-p53C’ is a complex made up of a transduction agent, TAT, and the therapeutic which is selected to reactivate dormant p53 anti-tumor peptides. Tumors have been shown to possess mutant or wild-type p53 alterations and can result in a malfunction of the cell cycle causing tumor growth and proliferation. In general, in vivo attempts are limited due to some CPPs having a short half-life, but RI-TAT is designed utilizing the properties of the D-isomer to prolong its half-life [152]. The in vitro studies compared colon cancer and lung carcinoma cells, which were treated with D-isomer RI-TAT-p53C’, L-Isomer RI-TAT-p53C’, and an untreated control [152]. After 7 days, the cell cycle was inhibited in these cancer cells treated with the D-isomer. Moreover, the in vivo studies provided promising results, showing that the mice that were treated with RI-TAT-p53C’ had an average tumor volume of 268 mm^3^ compared to the 573 mm^3^ average of the control group [152]. While reducing the average solid tumor volume by more than 50%, it extended the survival of the treated groups with an average survival period of 70 days compared to the untreated control, with an average of 11 days [152].

### 5.2. DTS-108 Trial

As described in Section 3, an important characteristic of CPPs is their ability to more efficiently and specifically deliver the therapeutic cargo to its specified location better than the anticancer drug alone. The utilization of CPPs has shown a significant reduction in toxicity due to the increased targeting capabilities of the CPPs. This was demonstrated in a pre-clinical trial using CPP DTS-108 to deliver an activatable cytotoxic drug called SN38 [156]. The DTS-108 SN38 conjugate was developed to bypass the hepatic activation required for SN38 to be released from other prodrugs, namely Irinotecan [156]. While Irinotecan is effective in treating colorectal cancers, it has a very low conversion rate from Irinotecan to SN38, resulting in high amounts of waste and difficulty in deciding dosage [156]. When combined with DTS-108, an increase from as low as 2% converted SN38 from Irinotecan to up to 29% free SN38 when combined with DTS-108 CPP, as well as a significant reduction in gastrointestinal cytotoxicity [156].

### 5.3. p28 Trial

As described above, p28 has both properties of tumor-preferential localization and intrinsic anti-tumor effects. p28 can activate p53 and inhibit the cancer cell cycle and induce apoptosis. Preclinical pharmacological studies of p28 provided significant evidence for efficacy without apparent toxicity or immunogenicity and prompted its entry into a phase I clinical trial. The primary objective of the first-in-class, first-in-human dose acceleration study was to determine the No Observed Adverse Effect Level (NOAEL) and maximum tolerated dose (MTD) of p28 in adult patients with advanced solid tumors. These patients had advanced tumors, which were unresponsive to traditional forms of treatment, and were also predicted to have around 6 months to live in their condition [148,149]. A total of 15 patients were administered p28 i.v. under an accelerated titration 3+3 dose escalation design. p28 was well tolerated with no significant adverse events and appeared to have anti-tumor activity in patients with advanced tumors.

Another phase I clinical trial of p28 as a single agent was conducted in pediatric patients with central nervous system (CNS) tumors [150]. Children with recurrent or progressive CNS tumors received p28 i.v. at 4.16 mg/kg/dose (the adult recommended phase II dose) using a rolling-6 study design. Similar results were found, although the adult p28 dose was tolerated in the adolescents, further showing that p28-based treatments can be handled among all age groups. The results of these trials have established that p28 is safe and well-tolerated at the recommended phase II dose (RP2D). Although p28 showed preliminary efficacy, the further development of this agent with other agents may prove more promising.

## 6. Current Limitations of CPPs

Despite the many advantages of CPPs, it should be noted that there are limitations of CPPs similar to any other agents. Cell selectivity, penetrating efficacy, and in vivo stability are considered as the major challenges for current CPPs. Many CPPs have low cell selectivity, probably due to their chemical characteristics (e.g., cationic amino acids). These types of CPPs need to be administrated directly to target tumors to avoid adverse effects. When CPPs are used in vivo, immunogenicity induced by CPPs may also limit their applications in clinics, similar to many other drug delivery carriers [157,158,159]. For CPPs as delivery carriers [160], not as peptide antigen vaccines [161,162], the assessment of immunogenicity is a critical step toward the characterization of clinical applicability.

Although it depends on the modes of entry and intracellular trafficking, endosomal uptake is another issue to increase efficacy and stability. The endosomal escape of CPPs can be improved by the addition of peptides that disrupt membranes at acidic pH as the pH in endosomes becomes acidic during endosomal maturation. Moreover, CPPs can be stabilized by chemical modifications to avoid inactivation by proteases. Recent advances in peptide chemistry will overcome such limitations in creating the next generation of CPPs.

## 7. Conclusions

To date, a major hurdle for cancer treatment is administering the desired drug efficiently while also leaving surrounding cells unharmed. Traditional treatments such as chemotherapy induce high rates of unwanted toxicity, because of their low specificity, and CPPs offer a new way of infiltrating the cell biome in a more effective and less toxic way. As described in this review, CPPs come in a large range of conformations, chemical properties, and bonding capabilities, each offering its own set of advantages when treating certain diseases. We discussed how these CPP complexes are either isolated naturally or synthesized, as well as their potential uses in diagnostics or therapeutics. The control over components of the cell that CPP-based treatments offer can be further optimized by the inclusion of organelle targeting sequences, granting us the ability to interact with the cells at an even more precise level. Several clinical trials show encouraging and promising results on how effective CPPs can be when combatting disease. In general, the selective delivery of cargoes (e.g., therapeutic and diagnostic agents) into an organ is also one of the major challenges for current drug development [163,164]. For instance, the central nervous system (CNS) is highly protected by several barrier structures, of which the blood-brain barrier (BBB) is the most critical one. This makes it very difficult to deliver drugs to the brain effectively. Some of CPPs successfully cross the BBB and deliver cargo molecules to the target site [59,165,166]. While more research is required to unlock the full potential of CPPs, CPP-based new approaches can ultimately lead to next-generation technologies as finely tuned vehicles for intracellular targeted delivery for cancer treatment.

## Figures and Tables

**Table 1:** Common CPPs from various sources and their applications.

Peptide Name	Sequence	Origin	Reference
TAT48-60	GRKKRRQRRRPPQ	TAT, Virus, HIV-1	[10,11,27,28]
Penetratin	RQIKIWFQNRRMKWKK	*Antennapedia Drosophila*, *Bee venom*	[29,30,31,32,33,34]
Maurocalcine	GDCLPHLKLCKENKDCCSKKCKRRGTNIEKRCR	*Scorpio Maurus Palmatus*, *Scorpion venom*	[35,36,37,38]
Pep-1	KETWWETWWTEWSQPKKKRKV	Pep-1, tryptophan dense NLS of Simian antigen	[39,40,41,42]
pVEC	LLILRRRIRKQAHAHSK	pVEC, Murine Vascular endothelial cadherin tissue	[25,43,44,45,46]
MAP	KLALKLALKALKAALKLA	Model Amphipathic Peptide (MAP)	[47,48,49,50]
Transportan	GWTLNSAGYLLGKINLKALAALAKKIL	Galanin–Mastoparan, a known chimeric peptide	[12,13,51,52]
CADY	GLWRALWRLLRSLWRLLWRA	ppTG11 derivative	[53,54,55]
Polyarginine	R_n_	Synthetic	[20,21,56,57,58]
p28	LSTAADMQGWTDGMASGLDKDYLKPDD	Bacteria, Azurin	[59,60,61,62,63,64,65,66,67,68,69]

Above is a short list of known and highly studied CPPs along with their respective origins and amino acid sequences. A comprehensive catalog of cell-penetrating peptides can be found at CPPSite 2.0 [9].

**Table 2:** CPPs in clinical trials.

Peptide	Cargo	Results	Reference
p28	p28	This shows that p28 can be tolerated by the body and did not result in any cytotoxic reactions. Shown to prevent p53 ubiquitination, thereby inhibiting cancer cell proliferation.	[148,149,150]
pVEC	Cyclic homing peptides CREKA CREKA_D_ AREKA Anti-cancer therapeutics Imaging/diagnostic tag	General CPPs (including pVEC and others in the above table) have low cancer-targeting abilities, but when combined with novel targeting sequences such as CREKA or AREKA, they can deliver therapeutics to the tumor.	[25,46,151]
ACCPs Activatable cell penetrating peptides	Fluorescence acceptors Fluorescence donors	When the ACCP comes in contact with matrix metalloproteinases (MMP-2 MMP-9) which are involved in metastasis and proliferation pathways, the fluorescing agent is activated, allowing for more accurate diagnostics.	[126,128]
RI-TAT	p53C’	Mice treated with the peptide complex showed a significant decrease in tumor numbers, and the proliferation of cancer cells is inhibited by the reactivation of p53	[152]
caPCNA	p21cip	caPCNA is a highly specific antigen that can interact with proteins along the DNA repair pathway inside cancer cells. Combined with the ability of p21 to induce apoptosis, the caPCNA-p21 complex is a promising treatment for breast cancer.	[153,154]
PepFect14 and 28	siRNAs	When combined with glioma-targeting peptides, PepFect showed highly efficient gene slicing of U87 GBM cells	[155]
SCPP-PS	Methotrexate disodium (MTX)	When mice with A549 lung tumors, MTX-SCCP-PS inhibits tumor growth and progression and improves survival time by an average of 37 days compared to free MTX	[139]
DTS-108	SN38	When compared to traditional chemotherapeutic agents such as irinotecan, DTS-108 introduced significantly higher levels of topoisomerase 1 inhibitor SN38	[156]

Listed are CPPs that have been involved in preclinical and/ or clinical trials and their respective results.

## Data Availability

Not applicable.
